# Genotype-by-environment interaction and stability of root mealiness and other organoleptic properties of boiled cassava roots

**DOI:** 10.1038/s41598-022-25172-8

**Published:** 2022-12-03

**Authors:** Kelechi Uchendu, Damian N. Njoku, Ugochukwu N. Ikeogu, Daniel Dzidzienyo, Pangirayi Tongoona, Samuel Offei, Chiedozie Egesi

**Affiliations:** 1 West Africa Centre for Crop Improvement (WACCI), University of Ghana, Accra, Ghana; 2 National Root Crops Research Institute (NRCRI), Umudike, Nigeria; 3 School of Integrative Plant Science, College of Agriculture and Life Sciences, Cornell University, Ithaca, NY USA; 4 International Institute of Tropical Agriculture (IITA), Ibadan, Nigeria

**Keywords:** Plant breeding, Plant genetics

## Abstract

Genetic enhancement of cassava aimed at improving cooking and eating quality traits is a major goal for cassava breeders to address the demand for varieties that are desirable for the fresh consumption market segment. Adoption of such cassava genotypes by consumers will largely rely not only on their agronomic performance, but also on end-user culinary qualities such as root mealiness. The study aimed to examine genotype × environment interaction (GEI) effects for root mealiness and other culinary qualities in 150 cassava genotypes and detect genotypes combining stable performance with desirable mealiness values across environments using GGE biplot analysis. Experiments were conducted using an alpha-lattice design with three replications for two years in three locations in Nigeria. The analysis of variance revealed a significant influence of genotype, environment, and GEI on the performance of genotypes. Mealiness scores showed no significant relationship with firmness values of boiled roots assessed by a penetration test, implying that large-scale rapid and accurate phenotyping of mealiness of boiled cassava roots remains a major limitation for the effective development of varieties with adequate mealiness, a good quality trait for direct consumption (boil-and-eat) as well as for pounding into ‘fufu’. The moderate broad-sense heritability estimate and relatively high genetic advance observed for root mealiness suggest that significant genetic gains can be achieved in a future hybridization program. The genotype main effects plus genotype × environment interaction (GGE) biplot analysis showed that the different test environments discriminated among the genotypes. Genotypes G80 (NR100265) and G120 (NR110512) emerged as the best performers for root mealiness in Umudike, whereas G13 (B1-50) and the check, G128 (TMEB693) performed best in Igbariam and Otobi. Based on the results of this study, five genotypes, G13 (B1-50), G34 (COB6-4), G46 (NR010161), the check, G128 (TMEB693), and G112 (NR110376), which were found to combine stability with desirable mealiness values, were the most suitable candidates to recommend for use as parents to improve existing cassava germplasm for root mealiness.

## Introduction

Cassava (*Manihot esculenta* Crantz) provides household food security and income for millions of smallholder farmers in sub-Saharan Africa (SSA)^1^, and is widely consumed as a cheap source of carbohydrates. It is a starch-storage root crop that is widely cultivated due to its remarkable ability to tolerate prolonged periods of drought, grow well in poor and acidic soils with less labour requirements than other crops, withstand biotic and abiotic stresses, produce reasonable yields under low soil fertility and erratic rainfall, and its adaptation to diverse agro-ecological conditions^2,3^. Furthermore, its flexibility in harvesting time enables the root crop to be stored naturally in the soil, making it a good famine reserve crop. Cassava is grown commercially on a large number of hectares across all agro-ecological zones of Nigeria and ranks first in the area under cultivation^4^. Because of the large expansion of the root crop across a wide range of agro-ecological conditions, it is very common to get different relative performances from the same genotypes when assessed in contrasting environments. The variations that occur in genotypic performance across environments are attributed to the effects of genotype × environment interaction (GEI), which is a common phenomenon in plant breeding programmes^2,5,6^.

GEI may be referred to as the differential phenotypic expression of genotypes under diverse environmental conditions, and one of its main effects is that it weakens the relationship between the values of the phenotype and genotype^7^. Various abiotic and biotic factors influence the expression of genes that control desirable agronomic and economic traits in cassava over the course of the crop’s growing period, which results in GEI^8^. For this reason, GEI has been a major focus for plant breeders. Breeders face the GEI challenge by evaluating genotypes in multi-environment trials (METs) to ensure that they select genotypes with ideal performance and adequate adaptability to target environments^9^. The adaptability of cassava genotypes to a wide range of environments can effectively be determined through rigorous statistical measures of the stability of individual genotypes. A genotype that is consistently well-ranked over multiple environments is said to be stable, and such genotype is considered to have broad or wide adaptation, whereas if stability is confined to a limited range, the genotype is deemed to have narrow or specific adaptation^10^.

Improvement of cassava has been primarily oriented towards important agronomic traits, especially yield and disease and pest resistance. This has led to the release of better yielding varieties that resist prevailing diseases and pests. Unfortunately, these varieties were poorly adopted by smallholder farmers because they lacked local consumption quality traits desired by end-users^11,12^. Studies have revealed that cassava farmers attach equal value to both agronomic performance and end-user culinary quality traits^13,14^. Therefore, new cassava varieties must have improved culinary qualities. In Nigeria, breeding efforts are focusing on the improvement of cassava cooking and eating quality traits, predominantly mealiness, to address the increasing demand for varieties that are suitable for the fresh consumption market segment. Adoption of such cassava varieties in Nigeria will largely rely not only on their yield and stress (abiotic and biotic) resistance performances, but also on organoleptic qualities and their suitability for processing into ready-to-eat foods. An accurate estimate of the stability of genotypes over time and space is therefore necessary for developing superior new cassava varieties with a potential high adoption rate among farmers and end-users. Various statistical models have been used to assess patterns of GEI, but among the most suitable approaches are those based on linear-bilinear models such as the genotype main effects plus genotype × environment interaction (GGE) biplot^15^.

Several studies have published information on the texture of boiled cassava roots, particularly with respect to its mealiness, but these did not cover multiple environments^16–20^. There are currently no reports of GEI effects on root mealiness and other organoleptic attributes of boiled cassava roots. In order to address this knowledge gap, the present study, therefore, evaluated 150 genotypes including one check variety for two years in three contrasting agro-ecological zones in Nigeria. Such information would present a new vista in designing breeding strategies for the development of cassava genotypes with enhanced sensory characteristics.

## Results

### Analysis of variance

The combined analysis of variance across environments showed that variances due to genotypes (G), environments (E), and GEI were highly significant (*P* < 0.001) for all the traits measured (Table 1). The analysis indicated that the G had the greatest effect on mealiness and colour, and accounted for 33.67% and 38.53%, respectively, of the total variation. The GEI had more impact on fibre (34.93%), adhesiveness (36.5%), softness (37.19%), taste (35.66%), aroma (40.37%), and firmness (32.05%) than the main effects of G and E. The E was the least contributor to variation for all the traits. The significant GEI effects, particularly for mealiness, prompted further investigation of the magnitude of genotype plus genotype × environment responses for this trait using GGE biplot analysis. The coefficient of variation (CV), which was used to measure variability among the traits, ranged from 11% for aroma to 29.35% for colour (Table 1).

**Table 1:** Mean square values and percentage sum of squares contribution to total variation for root mealiness and other culinary quality traits in 150 cassava genotypes phenotyped in three environments in Nigeria.

Source of variation	df	Mealiness	Fibre	ADH	Softness	Taste	Colour	Aroma	Firmness
Genotypes (G)	149	0.63***	0.25***	0.34***	0.42***	0.29***	1.66***	0.18***	2.67***
Environments (E)	2	13.29***	0.11***	3.21***	5.36***	1.31***	2.79***	2.01***	104.67***
Interactions (GEI)	298	0.27***	0.14***	0.26***	0.36***	0.19***	0.65***	0.21***	1.82***
%TSS due to G		33.67	30.23	23.99	21.73	27.10	38.53	16.80	23.57
%TSS due to E		9.46	0.18	3.04	3.73	1.62	0.87	2.56	12.39
%TSS due to GEI		28.41	34.93	36.50	37.19	35.66	30.31	40.37	32.05
Mean		1.32	1.76	1.88	2.45	2.19	1.96	2.31	3.77
CV (%)		29.33	13.84	16.78	14.95	12.00	29.35	11.00	24.48

df, Degrees of freedom; ADH, Adhesiveness; %TSS, Percentage of total sum of squares explained; CV, Coefficient of variation.

***Significant level at *P* < 0.001.

### Mean performance and stability of the genotypes

**Figure 1:**
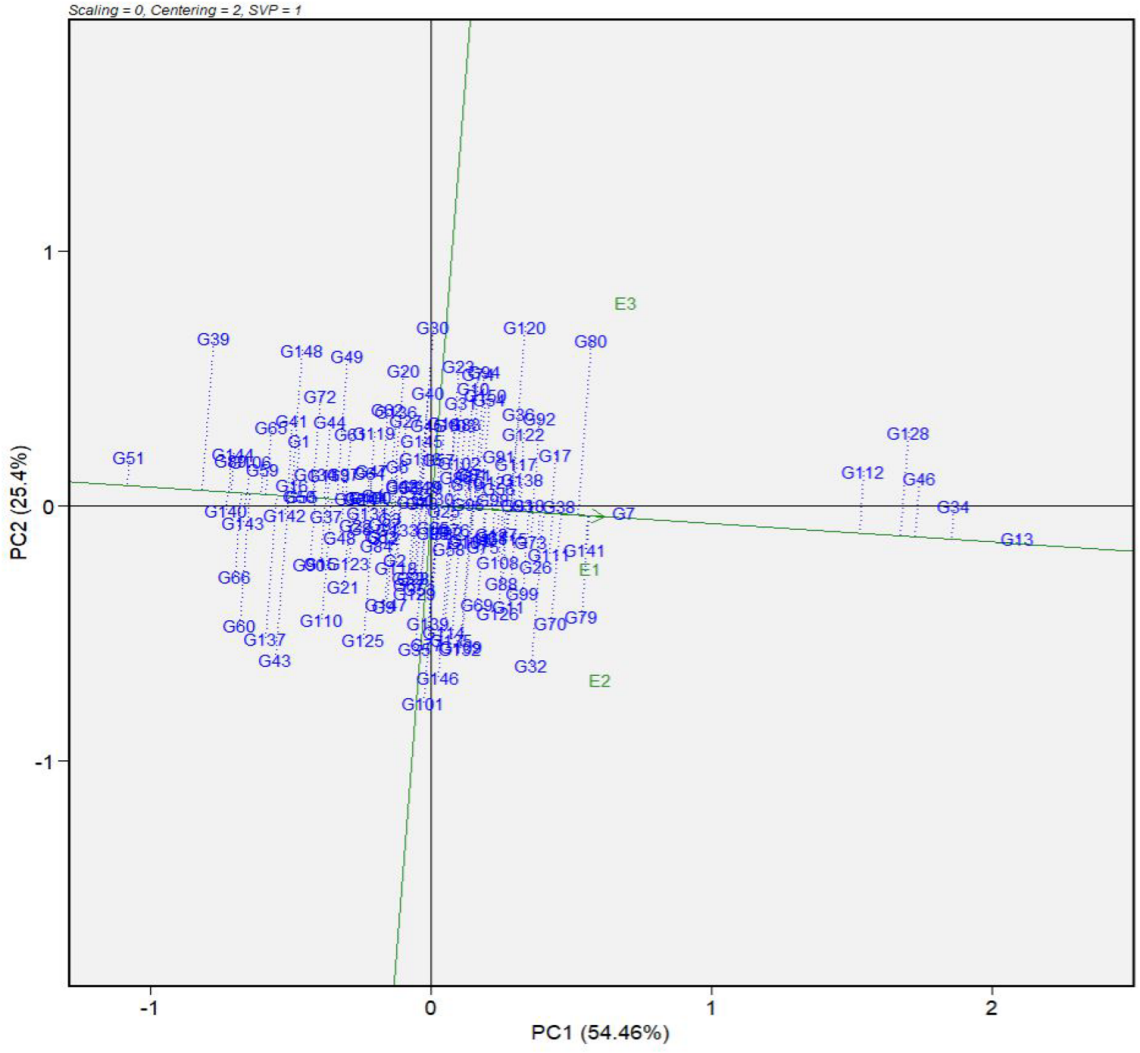
Mean performance versus stability pattern of the genotype main effects plus genotype × environment interaction (GGE) biplot based on the sensory mealiness data of 150 cassava genotypes grown in three environments in Nigeria. Environment names are coded as: E1, Igbariam; E2, Otobi; E3, Umudike. G1-G150 are genotypes defined in Supplementary Table S1.

From this section, results for root mealiness are presented using GGE biplots, as this was the focus trait. The partitioning of GGE through GGE biplot analysis showed that PC1 and PC2 accounted for 54.46% and 25.4% of the GGE sum of squares, respectively, explaining 79.86% of the total variation due to G + GEI for both years (Fig. 1). In this GGE biplot, genotypes were ranked along the average environment coordinate horizontal axis (AEC x-axis) with an arrow pointing to higher mean sensory scores across environments. Thus, genotypes trending towards the direction of G13 (B1-50), G34 (COB6-4), G46 (NR010161), the check, G128 (TMEB693), and G112 (NR110376) had desirable mealiness values, while genotypes in the opposite direction along the AEC x-axis such as G51 (NR050667) and G39 (CR35-10) produced boiled roots that were non-mealy. The stability of individual genotypes measured by the length of their projection from the AEC x-axis, showed that G13 (B1-50) was highly stable and also scored as the mealiest. Genotypes G7 (B1-23) and G38 (CR24-9) were equally stable, but were intermediate in mealiness. G51 (NR050667), though stable, had a low mealiness value compared to other genotypes evaluated in this study. Genotype G101 (NR110213) was low in mealiness and highly unstable (Fig. 1).

### Identifying best performing genotypes and mega-environments

**Figure 2:**
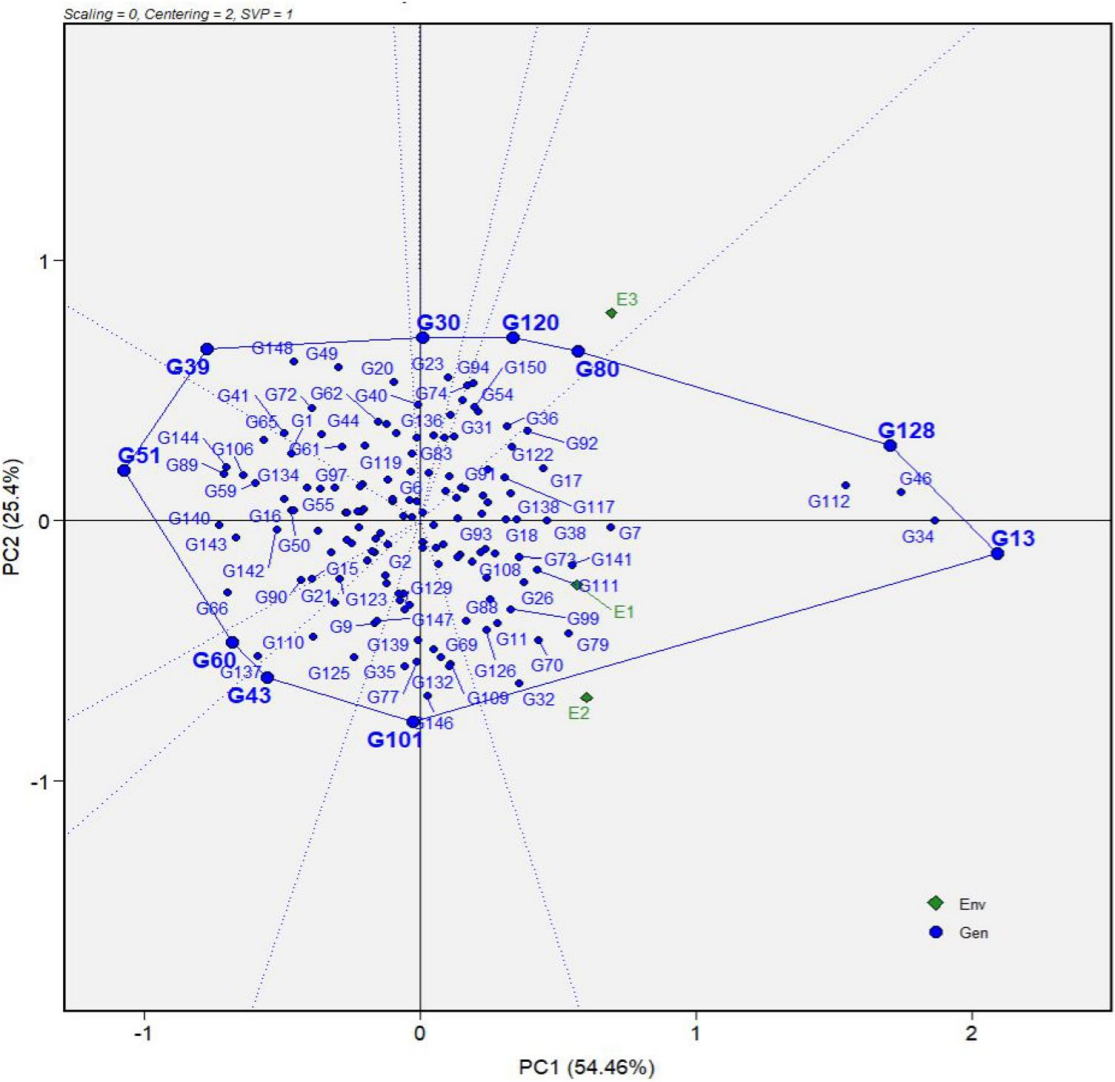
The polygon (which-won-where) view of the genotype main effects plus genotype × environment interaction (GGE) biplot based on the sensory mealiness data of 150 cassava genotypes grown in three environments in Nigeria. Environment names are coded as: E1, Igbariam; E2, Otobi; E3, Umudike. G1-G150 are genotypes defined in Supplementary Table S1.

The polygon view of the GGE biplot revealed the genotypes that had the best performance in specific environments (Fig. 2). G13 (B1-50) and the check, G128 (TMEB693), G30 (COB5-11), G39 (CR35-10), G43 (IBA083739), G51 (NR050667), G60 (NR070632), G80 (NR100265) and G120 (NR110512), and G101 (NR110213) located on the vertices of the polygon were the vertex genotypes and were thus considered as the most responsive genotypes to the environments. The other genotypes were contained within the polygon and were found less responsive in their respective directions. Genotypes G80 (NR100265) and G120 (NR110512) were identified as the best performers in environment E3 (Umudike), as they were grouped in one sector of the polygon. The winning genotypes in environments E1 (Igbariam) and E2 (Otobi) were G13 (B1-50) and the check, G128 (TMEB693). The pattern of environments in the biplot indicated the existence of two contrasting mega-environments. Thus, environment E3 (Umudike) formed the first mega-environment, while the second mega-environment had environments E1 (Igbariam) and E2 (Otobi) (Fig. 2).

### Discriminating ability and representativeness of test environments

**Figure 3:**
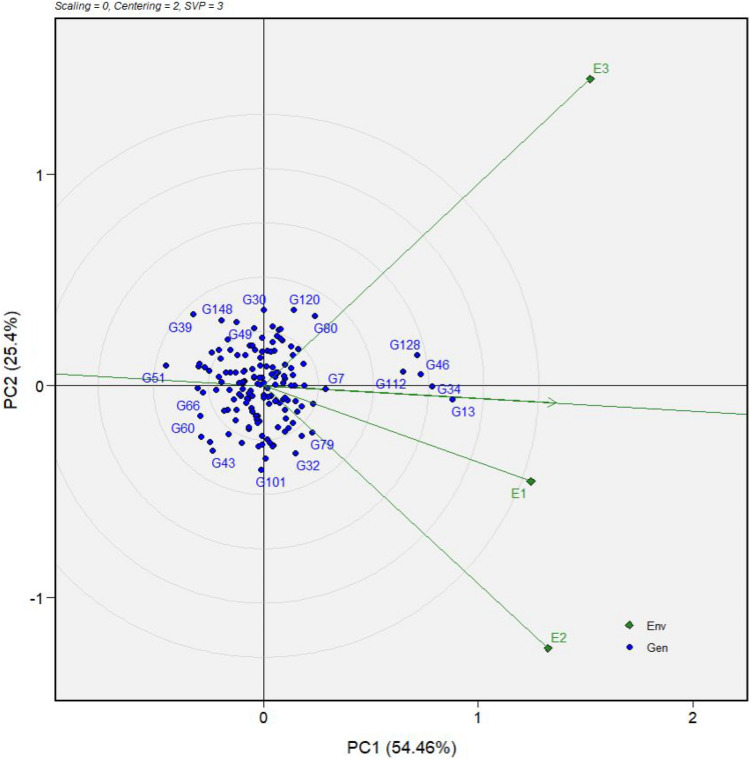
The average-environment coordinate (AEC) view of the genotype main effects plus genotype × environment interaction (GGE) biplot showing the discriminating ability and representativeness of the three test environments based on the sensory mealiness data of 150 cassava genotypes. Environment names are coded as: E1, Igbariam; E2, Otobi; E3, Umudike. G1-G150 are genotypes defined in Supplementary Table S1.

The concentric circles on the GGE biplot enabled the visualization of the length of the environment vectors (the lines that connect the test environments to the biplot origin), which is proportional to the standard deviation within the respective environments and is a measure of the discriminating power of the environments. Therefore, among the three environments, E3 (Umudike) had the longest vector and was adjudged as the most discriminating (informative), whereas E1 (Igbariam) was the least discriminating (non-informative), as indicated by the relative length of its vector (Fig. 3). Also, in biplot analysis, the test environment that has a smaller angle with the AEC x-axis is more representative of other test environments. Thus, environment E1 (Igbariam) was highly representative of other environments. Environments E3 (Umudike) and E2 (Otobi) were the least representatives (Fig. 3).

### Phenotypic correlations among traits

Results of the pairwise correlation analysis showed that softness (r = 0.46), taste (r = 0.48), colour (r = 0.15), and aroma (r = 0.26) had a positive significant (*P* < 0.01) correlation with mealiness (Table 2). In contrast, fibre (r = − 0.40) showed a negative significant (*P* < 0.01) relationship with mealiness. Also, fibre was negatively associated with a number of traits including softness (r = − 0.39), taste (r = − 0.12), colour (r = − 0.13), and aroma (r = − 0.15). Taste and aroma showed a moderate significant positive correlation (r = 0.40; *P* < 0.01). A lower magnitude of correlation coefficient was observed between softness and taste (r = 0.14; *P* < 0.01) as well as between adhesiveness and colour (r = 0.11; *P* < 0.05), taste and colour (r = 0.12; *P* < 0.05), softness and aroma (r = 0.10; *P* < 0.05), colour and aroma (r = 0.10; *P* < 0.05), and taste and instrumental firmness (r = 0.09; *P* < 0.05). On the other hand, adhesiveness was found to be significantly and negatively correlated with softness (r = − 0.10; *P* < 0.05) and instrumental firmness (r = − 0.15; *P* < 0.01). Similarly, instrumental measurements of firmness were negatively correlated to root softness (r = − 0.25; *P* < 0.01). However, there was no significant correlation between sensory scores of mealiness and firmness values of boiled roots assessed by penetration test (Table 2).

**Table 2:** Phenotypic correlation coefficients among end-user culinary quality traits phenotyped for 150 cassava genotypes in three environments in Nigeria.

Trait	Fibre	ADH	Softness	Taste	Colour	Aroma	Firmness
Mealiness	− 0.40**	0.02 ns	0.46**	0.48**	0.15**	0.26**	− 0.01 ns
Fibre	–	− 0.04 ns	− 0.39**	− 0.12**	− 0.13**	− 0.15**	0.09 ns
ADH		–	− 0.10*	0.02 ns	0.11*	− 0.02 ns	− 0.15**
Softness			–	0.14**	− 0.02 ns	0.10*	− 0.25**
Taste				–	0.12*	0.40**	0.09*
Colour					–	0.10*	0.08 ns
Aroma						–	− 0.02 ns

ADH, Adhesiveness; ns, Not significant.

*Significant level at *P* < 0.05.

**Significant level at *P* < 0.01.

### Genetic components and heritability estimates

The error variances for all the traits were slightly higher than their corresponding genotypic variances except for colour of boiled roots (Table 3). Broad-sense heritability estimates varied considerably for all the traits and were generally moderate for mealiness (0.46) and colour (0.52) (Table 3). Low heritability estimates were observed for fibre (0.33), adhesiveness (0.33), softness (0.31), taste (0.38), aroma (0.17), and firmness (0.36). The phenotypic coefficient of variation (PCV) ranged from 10.6% to 29.31%, with colour of boiled roots having the highest value, followed by mealiness (27.31%) and firmness (22.19%), while the lowest value was observed for aroma. Moderate PCV values (10–20) were observed for fibre, adhesiveness, softness, and taste. The genotypic coefficient of variation (GCV) varied from 4.33% for aroma to 21.04% for colour of boiled roots. The magnitude of the difference between the PCV and GCV values was moderate for all the traits measured (Table 3). The analysis of the expected genetic advance expressed as a percentage of mean (GAM) showed that root mealiness could be improved by 25.76%, whereas 9.39% progress could be made in the improvement of root softness. Also, aroma could be improved by 3.9%, while progress of 10.05% could be made in root taste (Table 3).

**Table 3:** Estimates of variance components, broad sense heritability, PCV, GCV, and genetic advance for eight traits in 150 cassava genotypes evaluated in three environments in Nigeria.

Trait	σ^2^g	σ^2^e	σ^2^p	H^2^	PCV (%)	GCV (%)	P-G	GA	GAM
Mealiness	0.06	0.07	0.13	0.46	27.31	18.56	8.75	0.34	25.76
Fibre	0.02	0.04	0.06	0.33	13.92	8.04	5.88	0.17	9.66
ADH	0.03	0.06	0.09	0.33	15.96	9.21	6.75	0.20	10.64
Softness	0.04	0.09	0.13	0.31	14.72	8.16	6.56	0.23	9.39
Taste	0.03	0.05	0.08	0.38	12.92	7.91	5.01	0.22	10.05
Colour	0.17	0.16	0.33	0.52	29.31	21.04	8.27	0.62	31.63
Aroma	0.01	0.05	0.06	0.17	10.60	4.33	6.27	0.09	3.90
Firmness	0.25	0.45	0.70	0.36	22.19	13.26	8.93	0.62	16.45

ADH, Adhesiveness; $\upsigma _{{\text{g}}}^{2}$, Genotypic variance; ${\upsigma }_{{\text{e}}}^{2}$, Residual variance; $\upsigma _{{\text{p }}}^{2}$, Phenotypic variance; H^2^, Broad sense heritability; PCV, Phenotypic coefficient of variation; GCV, Genotypic coefficient of variation; GA, Genetic advance; GAM, Genetic advance as a percentage of mean.

## Discussion

The significance of genotypes and environments main effects showed that some genotypes were stable across environments, whereas the significance of genotype × environment interaction indicated specific adaptation of some genotypes to certain environments. The highly significant genotype effects observed for all the traits measured showed that the genotypes constitute a pool of germplasm with a considerable amount of genetic variation. This genetic variability indicates that careful selection and hybridization among these contrasting genotypes may result in additional significant genetic gains in a cassava breeding programme in Nigeria aimed at improving the traits of interest. Environmental effects were highly significant for all the traits studied, indicating the presence of wide variation in testing conditions under which the genotypes were assessed. This suggests the need to conduct multi-environment trials to identify genotypes with wide and specific adaptation, as well as with the best performance for the traits. The highly significant GEI effects for all the traits showed differential genotypic performance in contrasting environments and also revealed changes in the mean performance of genotypes due to the environment. The GEI is a common occurrence in cassava as shown in the present study and which is corroborated by several other studies^21–25^, and justifies the need for multi-environment testing to identify and select best performers for specific environments^2^.

The root mealiness variation due to genotype was higher than the environmental influence on the trait, which suggests that mealiness in cassava is a complex trait influenced more by genotypic effects. This finding corroborates earlier reports in potato^26^ and cassava^17^. Moreover, the relatively high genotype and low environment effects on mealiness suggest that evaluation in fewer environments may be needed to discriminate genotypes with superior performance, stability and broad adaptation. This also suggests good prospects for enhancing cassava for the trait. However, the presence of a significant effect of GEI on mealiness indicates that some genotypes may fail to respond positively to improved conditions of the test environments. Hence, the need for a more definitive analysis to increase selection efficiency and facilitate the recommendation of superior new genotypes.

To detect the relative stability and GEI of promising genotypes, the applicability of GGE biplot analysis to data obtained from METs is of the utmost importance. The present study revealed that the GGE biplot was an effective statistical model for discriminating the cassava genotypes based on their mean performance and stability, identifying the best performing genotypes within a mega-environment, and evaluating the test environments for effective genotype evaluation based on their discriminating ability and representativeness^27^. With respect to the visual comparison of the genotypes based on both mean performance and stability across the test environments, the GGE biplot identified G13 (B1-50), G34 (COB6-4), G46 (NR010161), the check, G128 (TMEB693), and G112 (NR110376) as the genotypes that combined desirable mealiness values with moderate to high stability and were found to be the best performers for the trait. These genotypes showed superior performance and stability across the test environments. Projections from the AEC x-axis for most of the genotypes were longer for mealiness, indicating poor stability of the trait over time and space. The biplot also enabled visual comparison of the test environments and genotypes studied, as well as their interrelationships. The vertex genotypes appeared farthest from the biplot origin and were thus the most responsive genotypes to the closest environment(s) compared to others. These genotypes performed either the best or the poorest in some or all environments^28^. Nonetheless, different genotypes emerged as best performers in different environments. The genotypes G80 (NR100265) and G120 (NR110512) were identified as the best performers in Umudike. The winning genotypes in Igbariam and Otobi were G13 (B1-50) and the check, G128 (TMEB693). No environment fell into sectors with G30 (COB5-11), G39 (CR35-10), G43 (IBA083739), G51 (NR050667), G60 (NR070632), and G101 (NR110213) as the vertex genotypes, implying that these were not the best in any environment, but the poorest performers in some or all the environments. This pattern suggested that the test environments consisted of two different mega-environments for root mealiness, with Umudike separated as the first mega-environment and Igbariam plus Otobi grouped as the second mega-environment for the trait. This indicated that the genotypes could be successfully evaluated for mealiness of boiled roots in two locations, thus Umudike and either Igbariam or Otobi were identified as mega-environments for evaluating cassava genotypes in Nigeria. Furthermore, evaluating test environments to detect those that are both discriminating (informative) and representative is one of the key objectives of GGE biplot analysis. In this study, Igbariam was the most representative and moderately discriminating test environment, as indicated by its smaller angle with the AEC x-axis and the relative length of its vector, respectively. This indicated that the environment was best for identifying widely adapted genotypes. The remaining test environments (Umudike and Otobi) were the most discriminating and least representative, as they fell relatively far away from the AEC x-axis, indicating their usefulness in detecting better-performing genotypes with specific adaptation.

From the breeder’s point of view, correlations measure the intensity of association between traits, detect new parental combinations for variety development, improve selection efficiency, and identify trait measurement redundancy^29,30^. The significant negative correlation between fibre and other organoleptic attributes of boiled cassava roots (mealiness, softness, taste, colour, and aroma) suggests that genotypes with high fibre content may produce roots that are not acceptable for consumption as boiled cassava. This corroborates with the findings of Safo-Kantanka et al.^31^, who reported a negative correlation between fibre content and cooking quality of boiled cassava. The relationship observed between mealiness and softness, taste, colour, and aroma implies that these traits are imperative towards designing an efficient cassava breeding programme aimed at enhancing root mealiness. In our study, no significant correlation was found between sensory scores of mealiness and instrumental firmness of boiled roots, thus suggesting that the assessment of mealiness of boiled cassava roots by instrumental measurements may not be relied upon. This observation is contrary to earlier reports by Padonou et al.^19^ and Franck et al.^20^.

Estimates of genetic components in a breeding population can serve as a basis for comparing selection response and the expected level of progress^32^. A slightly larger proportion of the observed phenotypic variance for colour of boiled roots was accounted for by the genotypic variance, signifying inherent genetic variation for the trait. However, error variances for mealiness, fibre, adhesiveness, softness, taste, aroma, and firmness were slightly higher than their corresponding genotypic variances, indicating the effect of environment on the expression of these traits. The level of influence of the environment on any trait is indicated by the magnitude of the difference between the PCV and GCV values. The analysis of the magnitude of the variability among the traits studied showed a moderate difference between the PCV and GCV values, suggesting that the apparent variations in the varieties were not only genotypic, but also due to the influence of the environment. Broad-sense heritability values for all the traits measured were found to be relatively low-to-moderate. The Mealiness and colour of boiled roots had moderate heritability, implying that reliable selection for these traits can be achieved. The remaining traits had relatively low heritability estimates, which implies that the expression of these traits is influenced more by environmental factors. The heritability value for root softness in this study is comparable with the findings of Iragaba et al.^33^. Nonetheless, broad-sense heritability alone does not provide a full indication of genetic gain that can be made through selection because it includes both additive and non-additive variances^34^. It is important to calculate the genetic advance as a percentage of mean to ascertain the actual progress. Estimates of genetic advance as a percentage of mean (GAM) ranged from 3.9% for aroma to 31.63% for colour of boiled roots. This means that selecting the top 5% of the base population could result in progress of 3.9 to 31.63% over the population mean. Traits such as mealiness and colour of boiled roots had moderate heritability estimates and relatively high genetic advance, indicating that selection of genotypes based on these traits could result in significant selection gains in future generations.

## Conclusion

The present study showed significant variation between the cassava genotypes for all the traits studied. The observed GEI effects for all the traits led to variations in the average ranks of the genotypes in varying environments, thus justifying the need for multi-environment testing of the genotypes before effective selection can be made. The GGE biplot analysis revealed two distinct mega-environments for evaluating cassava genotypes in Nigeria and also identified G13 (B1-50), G34 (COB6-4), G46 (NR010161), the check variety, G128 (TMEB693), and G112 (NR110376) as the best performing genotypes for root mealiness. These genotypes were stable and adaptable across test environments and could be used as parental materials for further genetic improvement through hybridization in Nigeria. Instrumental measurements of firmness were not significantly correlated to sensory scores of mealiness and may therefore not be reliable in predicting the mealy texture of boiled cassava roots. The moderate broad-sense heritability and relatively high genetic advance obtained for root mealiness suggested potential for genetic improvement.

## Materials and methods

### Genetic material

A total of 150 cassava genotypes including the officially released white-fleshed poundable cassava (TMEB693), which served as check, were used for the study (Supplementary Table S1). The genetic material was sourced from the National Root Crops Research Institute (NRCRI) in Nigeria and consisted of both white and yellow-fleshed genotypes.

### Experimental sites

Trials for these genotypes were conducted over two cropping seasons (2018 and 2019) at three locations: Igbariam (5° 56′ N, 7° 31′ E; mean annual rainfall of 1800 mm; altitude of 150 m; mean annual temperature of 24–32 °C; Dystric Luvisol soils; forest savannah transition zone), Otobi (7° 20′ N, 8° 41′ E; mean annual rainfall of 1500 mm; altitude of 319 m; mean annual temperature of 24–35 °C; Ferric Luvisol soils; southern Guinea savannah zone), and Umudike (5° 29′ N, 7° 24′ E; mean annual rainfall of 2200 mm; altitude of 120 m; mean annual temperature of 22–31 °C; Dystric Luvisol soils; humid forest zone) in Nigeria. These locations represent the major cassava-growing agro-ecological zones in the country. In the present study, the sum of the two years in each location constituted a single environment. This gave a total of three test environments, that is, Igbariam 2018 and 2019 = E1, Otobi 2018 and 2019 = E2, and Umudike 2018 and 2019 = E3.

### Experimental design and management

Field trials were established at the onset of rains using a 10 × 15 alpha lattice design with three replications in each location. A replication consisted of 10 randomized incomplete blocks each containing 15 genotypes. Genotypes were planted as single rows of 5 plants with an inter-row spacing of 1 m and intra-row spacing of 0.8 m, making a basic plot size of 4 m^2^. Blocks were separated by 1.2 m alleys to minimize inter-block plant competition. No fertilizers or herbicides were applied; nonetheless, fields were kept clean by regular hand weeding. Harvesting was done in all the locations at 12 months after planting (MAP).

### Data collection

Data for individual genotypes were collected at 12 MAP on root mealiness and other quality attributes of boiled cassava roots, which include taste, softness, fibre, adhesiveness (ADH), aroma, colour, and firmness. The genotypes were phenotyped for these quality attributes using subjective estimates except for firmness of boiled roots, which was determined using a puncture force test.

### Boiled cassava sample preparation

Freshly harvested and healthy cassava roots were selected from each genotype, peeled and cut with a kitchen knife into roughly uniform-sized pieces. Samples for sensory analysis (10 root pieces) were washed twice, immersed in boiling water in enamel pots on a domestic gas cooker and left to cook for 25 min. The labelled boiled root samples were then removed from the pots and kept warm in an insulated box until ready to be served for sensory analysis.

### Descriptive sensory analysis

A panel of 10 assessors participated in the descriptive sensory test and evaluated the boiled root samples during the two consecutive years. These assessors were indigenes of the major cassava growing communities in Nigeria who regularly use cassava in their diets. They were recruited based on their interest and availability to participate. Mealiness, taste, softness, fibre, adhesiveness, aroma, and colour were the quality attributes of boiled cassava considered in this study. The assessors were thus trained to understand and quantify these attributes using numeric ratings based on hedonic scales, as described in Raji et al.^35^ with slight modification (Table 4). The attributes were scored after assessors tasted the samples. Ten samples (genotypes) were evaluated per session. Samples were presented to assessors in white plastic plates at room temperature, coded with random three-digit numbers. Boiled root samples were consumed plain. Water was provided to the assessors for mouth rinsing before and between tasting samples. Assessors performed the sensory test independently in separate tasting booths, with no interaction among assessors.

**Table 4:** Scales used in sensory analysis of boiled cassava roots.

Organoleptic attribute	Descriptor
Mealiness	0: Non mealy; 1: Fairly mealy; 2: Mealy; 3: Very mealy
Fibre	1: Low fibre; 2: Moderate fibre; 3: High fibre
Adhesiveness	1: Non sticky; 2: Slightly sticky; 3: Sticky
Softness	1: Very hard; 2: Hard; 3: Soft; 4: Very soft
Taste	1: Bitter; 2: Bland; 3: Sweet
Colour	1: Yellow; 2: Cream; 3: White
Aroma	1: Unpleasant; 2: Bland; 3: Pleasant

Hedonic scale: Mealiness, 0–3; Fibre, 1–3; Adhesiveness, 1–3; Softness, 1–4; Taste, 1–3; Colour, 1–3; Aroma, 1–3.

### Instrumental firmness analysis

An instrumental test was performed using a digital penetrometer (Model number: FHP-803, Vetus Industrial Company Limited, Hefei, China) to assess the firmness of boiled cassava roots. Three roots of each genotype were peeled, washed and cut into 3-cm-thick slices using a kitchen knife and ruler. For each genotype, three slices per root were randomly selected, immersed in boiling water in enamel pots on a domestic gas cooker and boiled for 25 min. Firmness was assessed by pushing the 7.9 mm diameter tip of the penetrometer to a final penetration depth of 1 cm into each boiled root slice. Firmness was defined as the peak force of penetration reached during the test. Two measurements were made on each sliced root. Each result was expressed as the mean value (in kg) of 18 readings.

### Data analysis

The effects of the genotype, environment, and genotype × environment interaction were determined for each attribute in an analysis of variance (ANOVA) using the standard linear model:

$$Y_{ijk} =\upmu +\upbeta _{{\text{i}}} + {\text{R}}_{{{\text{ij}}}} + {\text{G}}_{{\text{k}}} + \left( {\upbeta _{{\text{i}}} \times G_{{\text{k}}} } \right) + e_{{{\text{ijkm}}}}$$

where *Y*_*ijk*_ is the phenotypic value, μ is the grand mean, β_i_ is the effect of environment *i*, *R*_ij_ is the effect of block *j* in environment *i*, *G*_k_ is the effect of genotype *k*, (β_i_ × *G*_k_) is the genotype × environment interaction effect associated with environment *i* and genotype *k*, and *e*_*ijkm*_ is the residual term. Genotypes were fitted as fixed effects, whereas environments were considered random effects in the model. Broad-sense heritability (H^2^) was calculated as:

$${\text{H}}^{{2}} = \frac{{\sigma_{g}^{2} }}{{\sigma_{g}^{2} + { }\sigma_{e}^{2} }}$$

where $\sigma_{g}^{2}$ and $\sigma_{e}^{2}$ are the variance components for the genotype effect and the residual error, respectively. Genotypic coefficient of variation (GCV) and phenotypic coefficient of variation (PCV) were estimated according to Singh and Chaudhary^36^, such that:

$${\text{GCV}}\left( \% \right) = \frac{{\surd {\upsigma }_{{\text{g}}}^{2} }}{{{\ddot{\text{X}}}}}{ } \times 100$$

$${\text{PCV}}\left( \% \right) = \frac{{{ }\surd {\upsigma }_{{\text{p}}}^{2} }}{{{\ddot{\text{X}}}}}{ } \times 100$$

where ${\ddot{\text{X}}}$ is the grand mean.

Expected genetic advance, GA, was calculated as:

$${\text{GA}} = \left( K \right) \sigma_{A} H^{2}$$

where GA is the expected genetic advance, *K* is selection differential (2.06 at 5% selection intensity) and *σ*_*A*_ is phenotypic standard deviation.

Genetic advance as percentage of mean (GAM) for each attribute was estimated as:

$${\text{GAM}} = \frac{{{\text{GA}}}}{{{\ddot{\text{X}}}}}{ } \times 100.$$

Phenotypic correlation coefficients among the culinary attributes were calculated to investigate their relationships using the *corr.test* function of the psych package in R. To visually examine the patterns of genotype (G) and genotype × environment interaction (GEI) in the multi-environment trial data as well as identifying stable and high performing genotypes for root mealiness across the test environments, the first two principal components (PC1 and PC2) were used to construct GGE biplots using the “metan” R package developed by Olivoto^37^. The GGE biplots were used to graphically display genotype evaluation (mean vs. stability), test environment evaluation (discriminating ability vs. representativeness), mega-environment differentiation, and specific adaptation (which-won-where). Data were tester centered (G + GE) and non-scaled as described in Yan and Tinker^27^. All data analyses were performed using R statistical software version 4.0.3^38^.

## Supplementary Information

Supplementary Information.

## Data Availability

The datasets generated and/or analysed during the present study can be found in the Cassavabase repository (https://www.cassavabase.org/).
