# Supplementary material for: Genotype-by-environment interaction and stability of root mealiness and other organoleptic properties of boiled cassava roots
Source: Sci Rep. 2022 Dec 3;12:20909. doi: 10.1038/s41598-022-25172-8 (PMC9719563; doi:10.1038/s41598-022-25172-8)
Supplement: Supplementary file 1 — Supplementary Information. [file 41598_2022_25172_MOESM1_ESM.pdf]

TITLE

Genotype-by-environment interaction and stability of root mealiness and other organoleptic properties of boiled cassava roots

AUTHORS AND AFFILIATED INSTITUTIONS

Kelechi Uchendu (First author)

National Root Crops Research Institute (NRCRI), Umudike, Nigeria

West Africa Centre for Crop Improvement (WACCI), University of Ghana, Accra, Ghana

Damian N. Njoku

National Root Crops Research Institute (NRCRI), Umudike, Nigeria

Ugochukwu N. Ikeogu

School of Integrative Plant Science, College of Agriculture and Life Sciences, Cornell University, Ithaca, NY, USA.

Daniel Dzidzienyo

West Africa Centre for Crop Improvement (WACCI), University of Ghana, Accra, Ghana

Pangirayi Tongoona

West Africa Centre for Crop Improvement (WACCI), University of Ghana, Accra, Ghana

Samuel Offei

West Africa Centre for Crop Improvement (WACCI), University of Ghana, Accra, Ghana

***Chiedozie Egesi (Corresponding author)**

National Root Crops Research Institute (NRCRI), Umudike, Nigeria

School of Integrative Plant Science, College of Agriculture and Life Sciences, Cornell University, Ithaca, NY, USA.

International Institute of Tropical Agriculture (IITA), Ibadan, Nigeria

Email: cne22@cornell.edu

Supplementary Table S1. List of cassava genotypes used for the study.

Entry	Genotype	Code	Root flesh colour	Entry	Genotype	Code	Root flesh colour	Entry	Genotype	Code	Root flesh colour
1	AR14-4	G1	Cream	26	COB4-100	G26	Cream	51	NR050667	G51	White
2	AR1-45	G2	White	27	COB4-27	G27	Cream	52	NR060169	G52	Cream
3	AR1-82	G3	Yellow	28	COB4-77	G28	White	53	NR060242	G53	White
4	AR31-1	G4	Cream	29	COB5-01	G29	Yellow	54	NR060246	G54	White
5	AR9-45	G5	White	30	COB5-11	G30	White	55	NR060251	G55	Yellow
6	B1-19	G6	Yellow	31	COB5-17	G31	Yellow	56	NR060333	G56	Cream
7	B1-23	G7	Yellow	32	COB5-44	G32	Yellow	57	NR060789	G57	Cream
8	B1-25	G8	Yellow	33	COB5-86	G33	Yellow	58	NR070004	G58	Yellow
9	B1-26	G9	Cream	34	COB6-4	G34	Cream	59	NR070240	G59	Yellow
10	B1-29	G10	Yellow	35	COB7-197	G35	White	60	NR070632	G60	Yellow
11	B1-48	G11	Cream	36	CR14B-218	G36	Cream	61	NR090001	G61	White
12	B1-5	G12	Yellow	37	CR15B-3	G37	White	62	NR090088	G62	Cream
13	B1-50	G13	Cream	38	CR24-9	G38	Cream	63	NR090127	G63	Cream
14	B1-51	G14	Cream	39	CR35-10	G39	Cream	64	NR090142	G64	Yellow
15	B1-56	G15	Yellow	40	CR44-6	G40	Cream	65	NR090146	G65	White
16	B1-58	G16	Cream	41	CR528-26	G41	White	66	NR090162	G66	Yellow
17	B1-61	G17	Yellow	42	CR8A-22	G42	White	67	NR090176	G67	Cream
18	B1-67	G18	Yellow	43	IBA083739	G43	Yellow	68	NR090182	G68	Yellow
19	B1-78	G19	Cream	44	MM915280	G44	White	69	NR100018	G69	White
20	B1-95	G20	Yellow	45	MM961751	G45	Cream	70	NR100024	G70	White
21	B2-37	G21	Yellow	46	NR010161	G46	White	71	NR100077	G71	White
22	B4-6	G22	Yellow	47	NR010408	G47	Yellow	72	NR100106	G72	Yellow
23	B5-15	G23	Cream	48	NR050080	G48	Yellow	73	NR100112	G73	Cream
24	B5-19	G24	Yellow	49	NR050166	G49	Cream	74	NR100126	G74	White
25	COB1-163	G25	Cream	50	NR050362	G50	White	75	NR100196	G75	White

Supplementary Table S1. Continued.

Entry	Genotype	Code	Root flesh colour	Entry	Genotype	Code	Root flesh colour	Entry	Genotype	Code	Root flesh colour
76	NR100216	G76	White	101	NR110213	G101	White	126	NR1S1185	G126	Yellow
77	NR100225	G77	Cream	102	NR110223	G102	Cream	127	TME419	G127	White
78	NR100248	G78	Yellow	103	NR110228	G103	Cream	128	TMEB693	G128	White
79	NR100252	G79	Yellow	104	NR110232	G104	Cream	129	TMS010085	G129	Cream
80	NR100265	G80	White	105	NR110238	G105	Cream	130	TMS0101134	G130	Cream
81	NR100297	G81	White	106	NR110267	G106	Cream	131	TMS010169	G131	White
82	NR100325	G82	White	107	NR110270	G107	Cream	132	TMS010354	G132	White
83	NR100401	G83	Cream	108	NR110315	G108	White	133	TMS011097	G133	Yellow
84	NR100417	G84	Cream	109	NR110337	G109	Cream	134	TMS011368	G134	Yellow
85	NR100449	G85	Cream	110	NR110348	G110	White	135	TMS011412	G135	Yellow
86	NR100450	G86	Yellow	111	NR110372	G111	Yellow	136	TMS050128	G136	Yellow
87	NR100499	G87	Cream	112	NR110376	G112	Cream	137	TMS0501653	G137	Yellow
88	NR110031	G88	Yellow	113	NR110411	G113	Yellow	138	TMS050311	G138	Yellow
89	NR110044	G89	Yellow	114	NR110433	G114	Cream	139	TMS050540	G139	Cream
90	NR110079	G90	Cream	115	NR110439	G115	Yellow	140	TMS050752	G140	Yellow
91	NR110084	G91	Cream	116	NR110476	G116	White	141	TMS051570	G141	Yellow
92	NR110109	G92	Cream	117	NR110485	G117	White	142	TMS051600	G142	Yellow
93	NR110118	G93	White	118	NR110489	G118	Yellow	143	TMS051601	G143	Yellow
94	NR110160	G94	Cream	119	NR110490	G119	Cream	144	TMS051625	G144	Yellow
95	NR110165	G95	White	120	NR110512	G120	White	145	TMS070489	G145	Yellow
96	NR110169	G96	White	121	NR1S10064	G121	Cream	146	TMS070539	G146	Yellow
97	NR110176	G97	Cream	122	NR1S10097	G122	Cream	147	TMS070649	G147	Yellow
98	NR110178	G98	White	123	NR1S1018	G123	Cream	148	TMS30572	G148	White
99	NR110179	G99	Cream	124	NR1S1048	G124	Cream	149	TMS950211	G149	Yellow
100	NR110181	G100	Cream	125	NR1S1112	G125	Yellow	150	TMS961708	G150	White
